# Immunization with the Malaria Diversity-Covering Blood-Stage Vaccine Candidate *Plasmodium falciparum* Apical Membrane Antigen 1 DiCo in Complex with Its Natural Ligand *Pf*Ron2 Does Not Improve the *In Vitro* Efficacy

**DOI:** 10.3389/fimmu.2017.00743

**Published:** 2017-06-27

**Authors:** Holger Spiegel, Alexander Boes, Rolf Fendel, Andreas Reimann, Stefan Schillberg, Rainer Fischer

**Affiliations:** ^1^Fraunhofer Institute for Molecular Biology and Applied Ecology IME, Aachen, Germany; ^2^Institute for Phytopathology and Applied Zoology, Justus-Liebig University Giessen, Giessen, Germany; ^3^RWTH Aachen University, Institute for Molecular Biotechnology, Aachen, Germany; ^4^Indiana Biosciences Research Institute (IBRI), Indianapolis, IN, United States

**Keywords:** agroinfiltration, growth inhibition assay, plant molecular farming, *Plasmodium falciparum*, strain-transcending immune responses, surface plasmon resonance spectroscopy, calibration-free concentration analysis

## Abstract

The blood-stage malaria vaccine candidate *Plasmodium falciparum* apical membrane antigen 1 (*Pf*AMA1) can induce strong parasite growth-inhibitory antibody responses in animals but has not achieved the anticipated efficacy in clinical trials. Possible explanations in humans are the insufficient potency of the elicited antibody responses, as well as the high degree of sequence polymorphisms found in the field. Several strategies have been developed to improve the cross-strain coverage of *Pf*AMA1-based vaccines, whereas innovative concepts to increase the potency of *Pf*AMA1-specific IgG responses have received little attention even though this may be an essential requirement for protective efficacy. A previous study has demonstrated that immunization with a complex of *Py*AMA1 and *Py*RON2, a ligand with an essential functional role in erythrocyte invasion, leads to protection from lethal *Plasmodium yoelli* challenge in an animal model and suggested to extend this strategy toward improved strain coverage by using multiple *Pf*AMA1 alleles in combination with *Pf*Ron2L. As an alternative approach along this line, we decided to use *Pf*Ron2L in combination with three *Pf*AMA1 diversity covering variants (DiCo) to investigate the potential of this complex to induce more potent parasite growth inhibitory immune response in combination with better cross-strain-specific efficacy. Within the limits of the study design, the ability of the *Pf*AMA1 DiCo-Mix to induce cross-strain-specific antibodies was not affected in all immunization groups, but the DiCo–*Pf*Ron2L complexes did not improve the potency of *Pf*AMA1-specific IgG responses.

## Introduction

Malaria remains a major global health problem affecting >200 million people and killing more than 500,000 per year (1). An effective malaria vaccine is regarded as an essential component of any eradication strategy. *Plasmodium falciparum* apical membrane antigen 1 (*Pf*AMA1), a *Plasmodium* protein functionally involved in human erythrocyte invasion, is one of the leading blood-stage vaccine candidates. Many studies indicate that *Pf*AMA1-specific antibodies contribute to naturally acquired semi-immunity, so the capacity of this protein to induce parasite growth-inhibitory responses has been investigated in animals (2–6) and humans (7–10).

Both *in vitro* and *in vivo* studies show that antibody responses induced by single *Pf*AMA1 alleles achieve significantly lower efficacy against heterologous strains (11). Several epidemiological studies in different countries have revealed large numbers of different *Pf*AMA1 haplotypes even in defined endemic areas (12, 13). This high degree of polymorphism in the field is likely to be a parasite strategy to evade the immune system (11), thus presenting a serious challenge for the development of effective *Pf*AMA1-based vaccine candidates. The problem has been tackled by different groups using either mixtures of up to seven *Pf*AMA1 alleles (14–18) or by the design of three so-called diversity covering (DiCo) variants (19). These artificial sequences were generated based on the analysis of over 300 different *PfAMA1* sequences from field isolates, and cover 97% of the observed amino acid variability affecting around 10% of the amino acid residues. Additionally, all potential N-glycosylation sites were removed using preferentially natural occurring mutations. Both strategies are successful in eliciting antibodies with a broader range of specificity by focusing the immune response toward conserved regions of the molecule. The outcome of several studies performed with *Pf*AMA1-based vaccines in animals and humans suggest that besides cross-strain efficacy, also the potency of the immune IgG needs to be improved to induce sufficient protection. Since variations in dose, adjuvant, formulation [protein in adjuvant, DNA, viral vectored as well as combinations thereof (20–22)] have shown only moderate improvements, it is believed that alternative strategies are required to improve the potency of *Pf*AMA1-specific antibodies.

*Plasmodium falciparum* apical membrane antigen 1 plays an important role in the erythrocyte invasion machinery and an essential step during invasion is the formation of a moving junction between the merozoite and the erythrocyte membrane. During this process, the connection between the two cells is maintained by the interaction between *Pf*AMA1 located on the surface of the parasite and *Pf*Ron2, another *P. falciparum* protein, which is translocated to the erythrocyte membrane early in the process (23–25). Previous studies have shown that antibodies (26–28), peptides (29, 30), and drugs (31, 32) that interfere with the AMA1–Ron2 interaction in different plasmodium species inhibit the growth of the parasite. Additionally, structural analysis of the *Pf*AMA1–*Pf*Ron2 complex has revealed extensive conformational changes in the *Pf*AMA1 variable loops surrounding the *Pf*Ron2-binding pocket compared to *Pf*AMA1 alone (25, 33–35) making those regions particularly interesting as targets for potent parasite growth-inhibitory antibodies. A complex of AMA1 and Ron2L (a synthetic peptide, representing the *Pf*AMA1-binding domain of *Pf*Ron2) as the immunogen achieved higher efficacy than AMA1 alone in an *in vitro* parasite growth inhibition assay (GIA) using *P. falciparum* and also protected mice against a lethal challenge with *P. yoelii* (35). Even though this strategy improves the potency of AMA1-based vaccines, it does not address the need for cross-strain protection, leading the authors of the abovementioned study to suggest the use of multiple *Pf*AMA1–*Pf*Ron2 complexes representing different *Pf*AMA1 alleles (35). As an alternative, we chose to investigate a scenario involving the minimum number of different recombinant molecules by using the three DiCo *Pf*AMA1 variants (19) in a complex with *Pf*Ron2L.

## Materials and Methods

### Bacteria, Plants, and Parasites

*Agrobacterium tumefaciens* strain GV3101:pMP90RK (GmR, KmR, RifR) (36) was used for the production of recombinant proteins in *Nicotiana benthamiana* plants by agroinfiltration. Parasite strains *P. falciparum* 3D7, FCR3, and HB3 (MR4, Manassas, VA, USA) were used for the GIAs.

### Construct Cloning and Transient Expression in *N. benthamiana*

The DiCo1-3 sequences (Figure S1 in Supplementary Material) were amplified from their source constructs (37) and introduced into the plant expression vector pTRAkc-ERH (linearized with *Nco*I/*Not*I) in-frame with an upstream signal peptide sequence and a downstream His_6_ tag and SEKDEL signal for retention in the endoplasmic reticulum (38). Additionally, six different alleles of *Pf*AMA1 (*Pf*AMA1-3D7, *Pf*AMA1-FCR3, *Pf*AMA1-HB3, *Pf*AMA1-Dd2, *Pf*AMA1-7G8, and *Pf*AMA1-RO33) were obtained as synthetic genes codon optimized for *N. benthamiana* from Geneart (Thermo Fisher Scientific, Waltham, MA, USA) and introduced into the plant expression vector pTRAkc-ERH using the cloning strategy mentioned above. All cloning steps were confirmed by DNA sequencing. The transformation and cultivation of *A. tumefaciens* as well as transient expression in *N. benthamiana* plants was carried out as previously described (38).

### Purification of Recombinant Proteins

The three DiCo variants and six *Pf*AMA1 alleles (*Pf*AMA1-3D7, *Pf*AMA1-FCR3, *Pf*AMA1-HB3, *Pf*AMA1-Dd2, *Pf*AMA1-7G8, and *Pf*AMA1-RO33) were purified by immobilized metal affinity chromatography (IMAC) followed by size exclusion chromatography (SEC) on a Superdex 75 column (GE Healthcare Life Sciences, Little Chalfont, UK) as previously described (39).

### Analysis of DiCo–*Pf*Ron2L Complex Formation

The concentration of *Pf*Ron2L (DITQQAKDIGAGPVASCFTTRMSPPQQICLNSVVNTALS), purchased in the oxidized form from Pepscan (Lelystad, The Netherlands) required for equilibrium saturation of the three purified DiCo molecules was determined by surface plasmon resonance (SPR)-based competition analysis using a Biacore T200 instrument (Biacore, Uppsala, Sweden). We mixed 796 nM of each purified DiCo variant with serial 1:3 dilutions of *Pf*Ron2L starting at 12.5 µM and descending to a minimum of 5.7 nM. The residual binding of the remaining free DiCo molecules was quantified using an S-series streptavidin chip coated with biotinylated *Pf*Ron2L.

### Formulation of Antigens

Immunization with DiCo-Mix (group D), DiCo-Mix, and *Pf*Ron2L at different injection sites (group D + R) or the Dico-Mix–*Pf*Ron2L complex (group C) was achieved using different vaccine dose formulations. The purified DiCo variants and the *Pf*Ron2L peptide were lyophilized (PBS, pH 7.4) in single-dose scale glass vials and stored at −20°C. For immunization, the lyophilized proteins were reconstituted in sterile water. To facilitate complex formation, the DiCo–*Pf*Ron2L mixture was incubated for 30 min at room temperature prior to final formulation with the adjuvant.

### SDS-PAGE and Immunoblot Analysis

Proteins were separated on 4–12% (w/v) NuPage polyacrylamide gradient gels (Thermo Fisher Scientific, Waltham, MA, USA) and either stained with Coomassie Brilliant Blue or transferred onto a nitrocellulose membrane (Whatmann, Dassel, Germany) for immunoblot analysis as previously described (39).

### Rabbit Immunization and IgG Purification

Rabbits were housed, immunized, and sampled by Biogenes GmbH (Berlin, Germany) according to national animal welfare regulations. Four rabbits were immunized with either DiCo-Mix (D, 50 µg), or DiCo-Mix (50 µg) and *Pf*Ron2L (50 µg) at different injection sites (D + R), or the Dico-Mix–*Pf*Ron2L complex (C, prepared by mixing 50 µg of DiCo-Mix with 50 µg *Pf*Ron2L), each formulated with the Biogenes proprietary adjuvant, on days 0, 28, and 56. Serum samples were collected on day 70. IgG purification and quantification was carried out as previously described (39).

### Analysis of Immune Sera

Antibody titers against the different *Pf*AMA1 variants were determined by direct-coating enzyme-linked immunosorbent assay (ELISA) as previously described (39), using DiCo-Mix, single DiCo variants, and six different *Pf*AMA1 alleles (*Pf*AMA1-3D7, *Pf*AMA1-FCR3, *Pf*AMA1-HB3, *Pf*AMA1-Dd2, *Pf*AMA1-7G8, and *Pf*AMA1-RO33). To measure the avidity of the immune sera for the DiCo-Mix, we used the NaSCN displacement ELISA, an adapted protocol called Avidity ELISA (40). Based on previous titer determinations, all serum samples were diluted to OD_405 nm_ = 0.6–0.8. The avidity index is the molar NaSCN concentration at which 50% of the bound serum antibodies can be eluted.

### Calibration-Free Concentration Analysis (CFCA) of Purified Rabbit Immune IgG

Antigen-specific antibody concentrations were measured in the purified rabbit antibody preparations by CFCA (41) using a Biacore T200 instrument. The antigens (DiCo-Mix, *Pf*AMA1-3D7, *Pf*AMA1-FCR3, and *Pf*AMA1-HB3) were separately covalently coupled to CM5 S-Series sensor chips using standard EDC–NHS chemistry as previously described (39).

### SPR-Based Competition Analysis

To confirm the CFCA results, a competition assay was carried out using the Biacore T200 instrument and the DiCo-Mix surface. To determine the quantity of DiCo and allele-specific antibodies, the purified IgG preparations were mixed either with running buffer, or a molar excess of DiCo-Mix, single DiCo (1-3), *Pf*AMA1-3D7, *Pf*AMA1-FCR3, or *Pf*AMA1-HB3, as well as a mixture of three alleles (*Pf*AMA1-3D7, *Pf*AMA1-FCR3, *Pf*AMA1-HB3) and a mixture of six alleles (*Pf*AMA1-3D7, *Pf*AMA1-FCR3, *Pf*AMA1-HB3, *Pf*AMA1-Dd2, *Pf*AMA1-7G8, and *Pf*AMA1-RO33). The 90-s injections were conducted under mass transport limitation, and the DiCo-Mix surface was regenerated between injections using 20-s pulses with 30 mM HCl. The binding signal resulting from competition mixtures was normalized against the end-point values for the corresponding buffer controls, which were set to 100%.

### Parasite Culture and GIA

*P. falciparum* strains 3D7A, HB3, and FCR3 were cultured under routine culture conditions and synchronized as described before (42). The ability of purified polyclonal rabbit IgGs to inhibit the growth of *P. falciparum* strains 3D7A, FCR3, and HB3 was determined by conducting GIAs as previously described (43, 44). Highly synchronous parasites were treated with eight serial dilutions of the rabbit IgG (1:2, starting at a final concentration of 6 mg/ml) at schizont stage. The parasites cultures were harvested at 42–44 h of coculture. As controls, BG98 (positive control, kindly provided by Ed Remarque, BPRC, Rijswijk, Netherlands) (45) and purified IgG from non-immunized rabbits (negative control) were used at a concentration of 6 mg/ml. Parasite growth was estimated using the pLDH-assay (42).

### Statistical Analysis

Titers, avidities, allele-specific antibody concentrations, competition data, and GIA IC_50_ values derived from the three different immunization groups (D, D + R, and C) were compared by one-way analysis of variance (ANOVA) using Origin data analysis software (OriginLab, Northampton, MA, USA). GIAs were analyzed using GraphPad Prism software package v7.02. For the determination of IC_50_-values, the growth curves were fitted using a 4-parameter logistic curve fit and the IC_50_-value estimated using the Hill equation. The level of statistical significance for all analyses was set at 0.05.

## Results

### Transient Expression and Purification of *Pf*AMA1 Variants

After generating the expression constructs and the corresponding recombinant *A. tumefaciens* cultures, small-scale transient expression was carried out in *N. benthamiana* allowing the provision of recombinant proteins within a few days (38). All three DiCo proteins (DiCo1-3), as well as the six alleles (*Pf*AMA1-3D7, *Pf*AMA1-FCR3, *Pf*AMA1-HB3, *Pf*AMA1-Dd2, *Pf*AMA1-7G8, and *Pf*AMA1-RO33) accumulated to high levels and were successfully purified from leaf tissue by IMAC and SEC.

### Analysis of DiCo–*Pf*Ron2L Complex Formation

**Figure 1:**
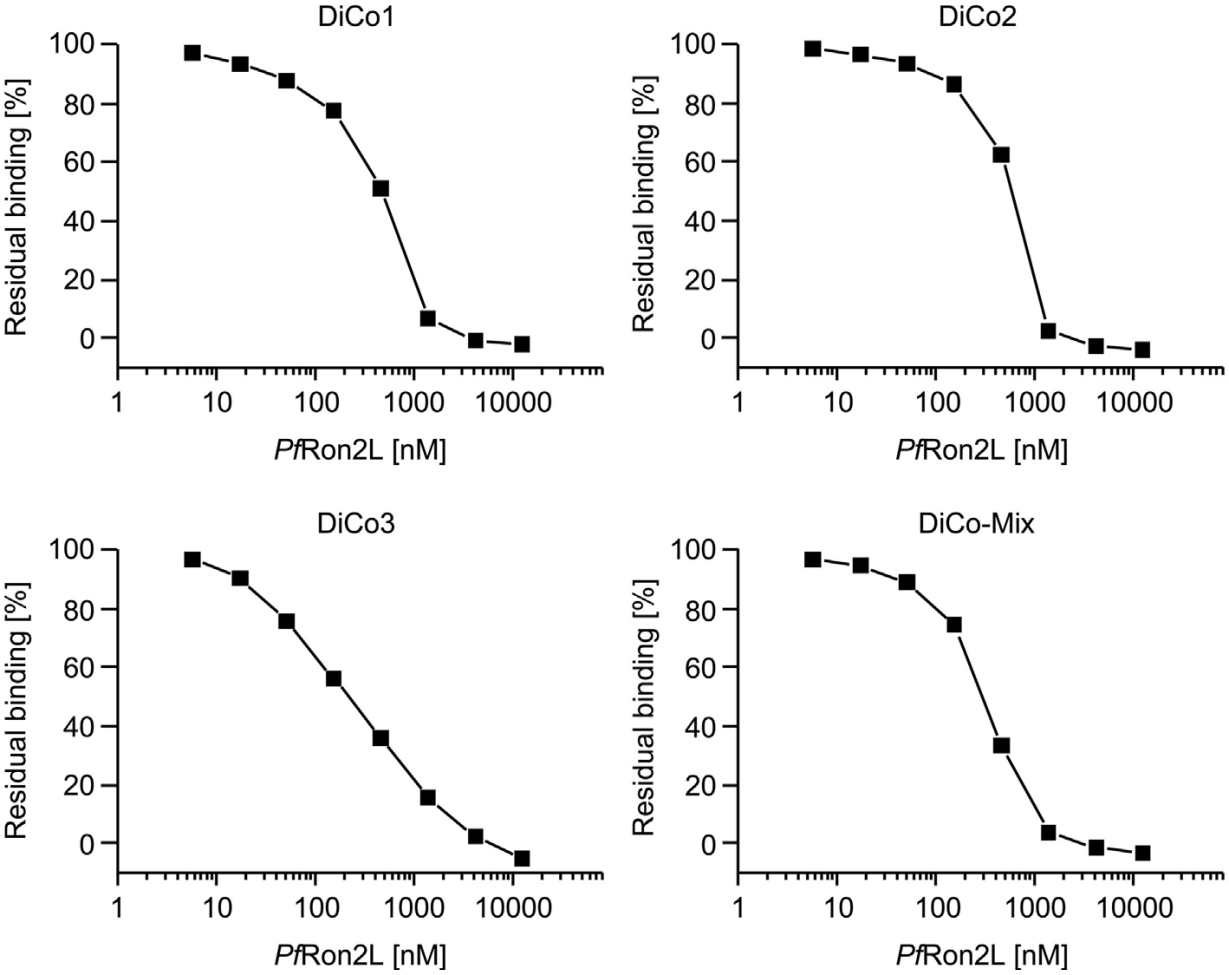
Analysis of DiCo–*Pf*Ron2L complex formation. To determine the equilibrium saturation of the DiCo–*Pf*Ron2L complex, surface plasmon resonance-based competition experiments were conducted using a biotinylated *Pf*Ron2L peptide immobilized on a Series S sensor chip streptavidin. Equivalent concentrations of purified DiCo1, DiCo2, DiCo3 as well as a balanced DiCo-Mix were mixed 1:1 with buffer (reference) or decreasing concentrations of the *Pf*Ron2L peptide. The final concentration in the competition assays was 50 µg/ml for the DiCo proteins with a molecular weight of approximately 62.5 kDa (796 nM) and 50 µg/ml (12,500 nM)—0.022 µg/ml (5.7 nM) for the *Pf*Ron2L peptide. The competition samples were incubated for 4 h at room temperature and the residual binding to the immobilized *Pf*Ron2L was analyzed. The buffer control (reference) was set to 100%, and residual binding was expressed as a percentage compared to the control.

The full equilibrium saturation of each purified DiCo variant as well as the DiCo-Mix was achieved at 12.5 µM (50 µg/ml) *Pf*Ron2L as illustrated by the complete reduction of the binding signal in SPR measurements (Figure 1). Although almost full saturation was achieved at a concentration >1,000 nM for DiCo1, DiCo2, and DiCo-Mix, a higher concentration was required for the equilibrium saturation of DiCo3 (>10,000 nM), probably reflecting the lower *Pf*Ron2L-binding affinity of this variant. The DiCo concentration (796 nM or 50 µg/ml) used for equilibrium saturation analysis was identical to the conditions used for the constitution of the immunization complex (50 µg DiCo-Mix plus 50 µg *Pf*Ron2L).

### Rabbit Immunizations and Characterization of Immune Sera

**Figure 2:**
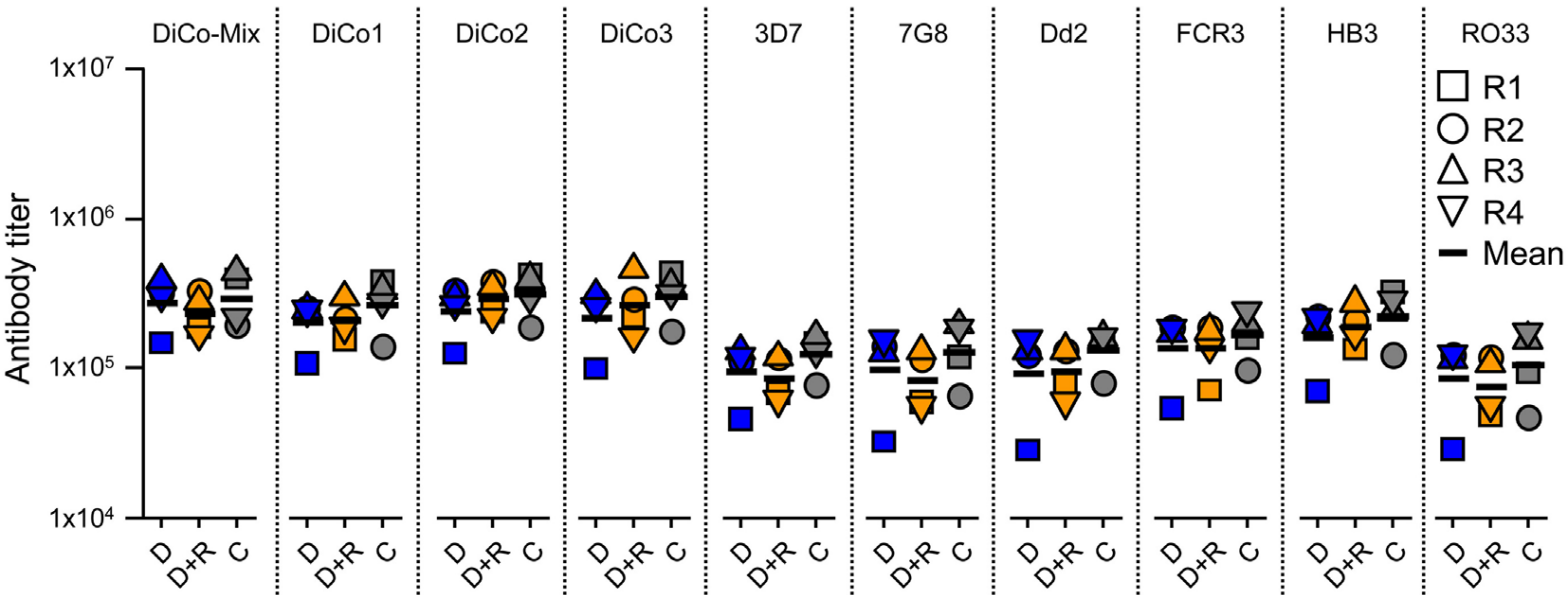
Determination of antibody titers in the serum samples. Four rabbits (R1–R4) in each group were immunized using a one prime (day 0) and two boost (day 28 and day 56) immunization schedule and the serum samples were collected on day 70. The antibody titers were assessed by direct-coating enzyme-linked immunosorbent assay using the DiCo-Mix and single DiCo variants as well as six different *Plasmodium falciparum* apical membrane antigen 1 alleles as coating antigens (indicated above each lane). D: group of rabbits that received 50 µg DiCo-Mix; D + R: group of rabbits that received 50 µg DiCo-Mix and 50 µg of *Pf*Ron2L peptide at different injection sites; C: group of rabbits that were immunized with the DiCo–*Pf*Ron2L complex formed by mixing 50 µg of DiCo-Mix with 50 µg of *Pf*Ron2L. To facilitate complex formation, the DiCo–*Pf*Ron2L mixture was incubated for 30 min at room temperature before formulation with the adjuvant and immunization. The end-point titers were defined as the highest dilution that gave double the value of the background (pre-immune serum).

**Figure 3:**
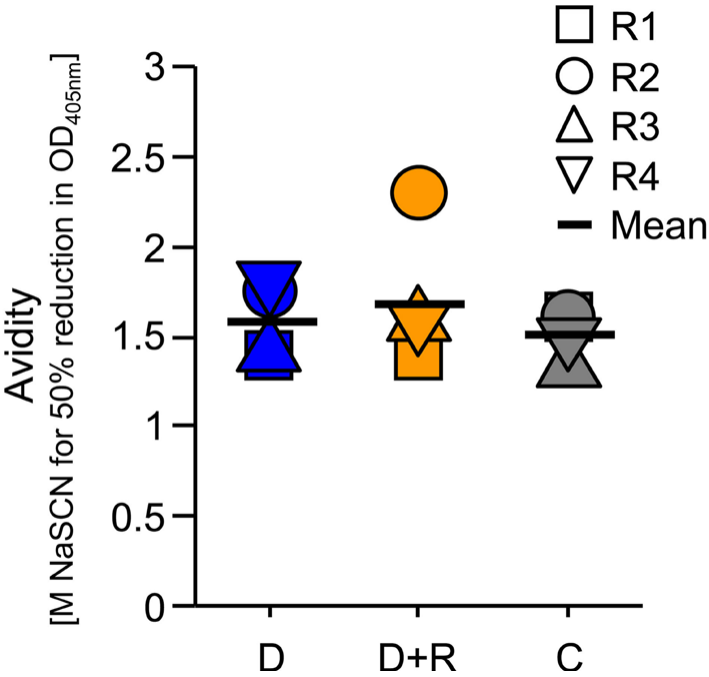
Determination of antibody avidity in the serum samples. The antibody avidity for the DiCo-Mix was assessed using the NaSCN-displacement enzyme-linked immunosorbent assay protocol and is defined as the NaSCN concentration (molar) required to reduce the OD_405 nm_ by 50% compared to the reference sample incubated without NaSCN. D: group of rabbits that received 50 µg DiCo-Mix; D + R: group of rabbits that received 50 µg DiCo-Mix and 50 µg of *Pf*Ron2L peptide at different injection sites; C: group of rabbits that were immunized with the DiCo–*Pf*Ron2L complex formed by mixing 50 µg of DiCo-Mix and 50 µg of *Pf*Ron2L. To facilitate the complex formation, the DiCo–*Pf*Ron2L mixture was incubated for 30 min at room temperature before formulation with the adjuvant and immunization.

Serum samples collected after the immunization of rabbit groups were analyzed by ELISA to determine specific IgG titers for DiCo-Mix, the three individual DiCo variants, and the different *Pf*AMA1 alleles (Figure 2). No significant differences in IgG titer were observed among the three groups (D, D + R, and C). Geometric mean titers against DiCo-Mix and the individual DiCo variants were approximately 2.5 × 10^5^ (Figure 2). Immune sera were also compared by avidity ELISA, revealing no significant differences among the immunization groups (Figure 3).

### Quantification and Analysis of Purified Immune IgG

**Figure 4:**
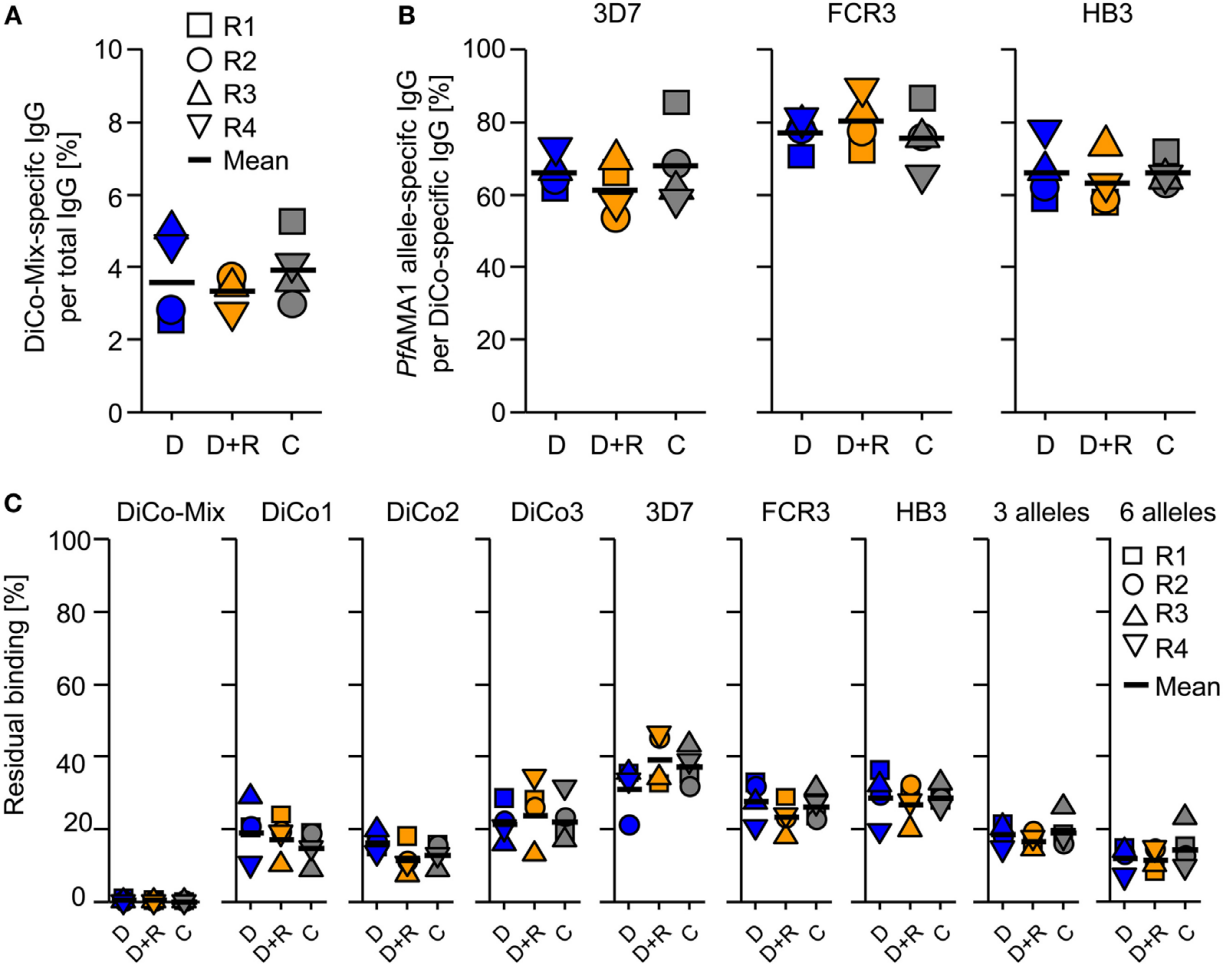
Quantification and analysis of purified immune IgG. The total IgG concentration in the purified IgG preparation was determined by analytical size exclusion chromatography. The antigen-specific IgG concentration for the immunization antigen (DiCo-Mix) as well as for three *Plasmodium falciparum* apical membrane antigen 1 (*Pf*AMA1) alleles (3D7, FCR3, and HB3) was quantified by surface plasmon resonance (SPR) spectroscopy (Biacore T200) using the calibration-free concentration analysis (CFCA) module. The DiCo-specific IgG concentration is expressed as a percentage of total IgG **(A)** and as an indication for balanced *Pf*AMA1 allele coverage as a percentage *Pf*AMA1 allele-specific response per DiCo-Mix-induced IgG response **(B)**. **(C)** SPR-based competition experiments were performed to confirm the CFCA results and to further characterize the purified IgG preparation after immunization with the different DiCo formulations (D: group of rabbits that received 50 µg DiCo-Mix; D + R: group of rabbits that received 50 µg DiCo-Mix and 50 µg of *Pf*Ron2L peptide at different injection sites; C: group of rabbits that were immunized with the DiCo–*Pf*Ron2L complex formed by mixing 50 µg of DiCo-Mix and 50 µg of *Pf*Ron2L). The DiCo-Mix was immobilized by EDC–NHS chemistry to a CM5 sensor chip. Purified IgG preparations were diluted 1:100 and mixed 1:1 with 30 µg/ml of each competitor antigen (indicated above each lane). For the DiCo-Mix and the three-allele mixture (3D7, FCR3, and HB3), the concentration of each antigen was 10 µg/ml, whereas in the six-allele mixture (3D7, FCR3, HB3, Dd2, 7G8, and RO33), the concentration of each individual *Pf*AMA1 allele was 5 µg/ml. Competition samples were incubated for 1 h at room temperature and residual binding to the DiCo-mix surface was determined. The reference (IgG sample mixed with buffer) was set to 100% and residual binding is expressed as a percentage compared to the reference.

Analytical SEC was used to determine the total quantity of IgG in the rabbit immune IgG purified by Protein A affinity chromatography. Sensor chips functionalized with DiCo-Mix, *Pf*AMA1*-*3D7, *Pf*AMA1*-*FCR3, or *Pf*AMA1*-*HB3 were used to determine the concentrations of antigen-specific antibodies in all preparations by CFCA. Table 1 shows the concentration of total IgG (milligrams per milliliter) and the quantity of antigen-specific antibodies indicated both by the concentration (milligrams per milliliter) and the proportion relative to total IgG (%). The quantity of allele-specific IgG is also shown relative to the quantity of DiCo-Mix-specific IgG (%). As already observed for the avidity index (Figure 3), there were no significant differences in immunogenicity among the three immunization groups (Figure 4A). Using the combination of *Pf*AMA1 and *Pf*Ron2L either as complex (C) or at separate injections sites (D + R) also had no quantitative effect on the strain specificity of the induced immune responses, which ranged from 60 to 70% (*Pf*AMA1-3D7 and *Pf*AMA1-HB3) up to 80% (*Pf*AMA1-FCR3) allele-specific IgG relative to the DiCo-Mix-specific immune IgG (Figure 4B). This result was also confirmed by SPR-based competition assays (Figure 4C). In the SPR-based competition assay using the DiCo-Mix surface, we also tested mixtures of three and six alleles to investigate the reactivity profile of the DiCo-Mix-specific antibody preparations. As also shown in Figure 4C, the use of DiCo-Mix as a competitor led to the complete abolition of binding, whereas three alleles (*Pf*AMA1-3D7, *Pf*AMA1-FCR3, *Pf*AMA1-HB3) neutralized 80% of the binding signal and six alleles (*Pf*AMA1-3D7, *Pf*AMA1-FCR3, *Pf*AMA1-HB3, *Pf*AMA1-Dd2, *Pf*AMA1-7G8, and *Pf*AMA1–RO33) neutralized 90% of the binding signal.

**Table 1:** Summary of total and antigen-specific antibody concentrations in the purified immune IgG preparations.

Antigen	ID	Total IgG (mg/ml)	DiCo-specific IgG (mg/ml)	DiCo-specific/total (%)	3D7-specific IgG (mg/ml)	3D7-specific/total (%)	3D7-specific/DiCo-specific (%)	FCR3-specific IgG (mg/ml)	FCR3-specific/total (%)	FCR3-specific/DiCo-specific (%)	HB3-specific IgG (mg/ml)	HB3-specific/Total (%)	HB3-specific/DiCo-specific (%)
DiCo-Mix	R1	13.35	0.34	2.55	0.21	1.57	61.76	0.24	1.80	70.59	0.20	1.50	58.82
DiCo-Mix	R2	17.69	0.50	2.83	0.32	1.81	64.00	0.39	2.20	78.00	0.31	1.75	62.00
DiCo-Mix	R3	15.44	0.78	5.05	0.52	3.37	66.67	0.62	4.02	79.49	0.52	3.37	66.67
DiCo-Mix	R4	13.63	0.63	4.62	0.46	3.37	73.02	0.51	3.74	80.95	0.49	3.59	77.78
DiCo-Mix + Ron2L	R1	14.01	0.50	3.57	0.33	2.36	66.00	0.36	2.57	72.00	0.29	2.07	58.00
DiCo-Mix + Ron2L	R2	15.50	0.58	3.74	0.31	2.00	53.45	0.45	2.90	77.59	0.34	2.19	58.62
DiCo-Mix + Ron2L	R3	15.25	0.53	3.48	0.37	2.43	69.81	0.44	2.89	83.02	0.39	2.56	73.58
DiCo-Mix + Ron2L	R4	13.79	0.38	2.76	0.22	1.60	57.89	0.34	2.47	89.47	0.24	1.74	63.16
DiCo-Mix–Ron2L complex	R1	16.86	0.89	5.28	0.76	4.51	85.39	0.77	4.57	86.52	0.64	3.79	71.91
DiCo-Mix–Ron2L complex	R2	18.01	0.54	3.00	0.37	2.05	68.52	0.41	2.28	75.93	0.34	1.89	62.96
DiCo-Mix–Ron2L complex	R3	17.15	0.62	3.62	0.38	2.22	61.29	0.47	2.74	75.81	0.40	2.33	64.52
DiCo-Mix–Ron2L complex	R4	14.17	0.58	4.09	0.34	2.40	58.62	0.38	2.68	65.52	0.38	2.68	65.52

### Growth Inhibition Assays

**Figure 5:**
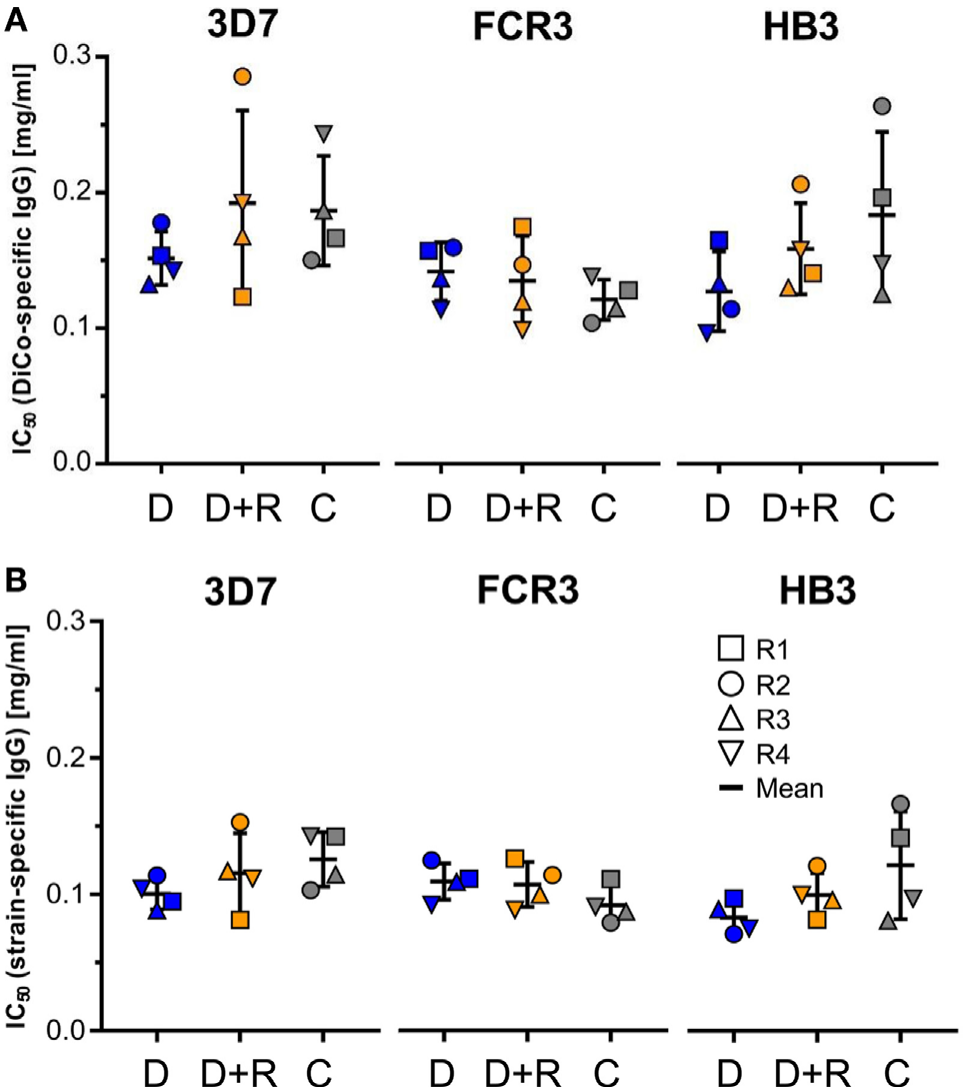
Growth inhibition assay. The parasite growth-inhibitory activity of the immune IgG preparations from the three different rabbit groups (see below) were assessed using three *P. falciparum* strains (3D7A, FCR3, and HB3). A serial dilution of purified IgG preparations starting at 6 mg/ml total IgG were used to determine IC_50_ values for the three *P. falciparum* strains. Based on the antigen-specific antibody concentrations (Table 1), IC_50_ values are expressed in micrograms per milliliter DiCo-specific IgG **(A)** and micrograms per milliliter allele-specific IgG **(B)**. D: group of rabbits that were vaccinated using 50 µg DiCo-Mix; D + R: group of rabbits that were vaccinated using 50 µg DiCo-Mix and 50 µg of *Pf*Ron2L peptide at different injection sites; C: group of rabbits that were immunized with the DiCo–*Pf*Ron2L complex formed by mixing 50 µg of DiCo-Mix and 50 µg of *Pf*Ron2L. To facilitate the complex formation, the DiCo–*Pf*Ron2L mixture was incubated for 30 min at room temperature before formulation with the adjuvant and immunization.

Growth inhibition assays were used to compare the ability of the immune IgG preparations derived from the three different rabbit groups (D, D + R, and C) to inhibit parasite growth. The data were used to calculate IC_50_ values for three different *P. falciparum* strains (3D7A, FCR3, and HB3) that have previously been used to characterize *Pf*AMA1 strain-specific antibodies. Figure 5 shows the IC_50_ values as normalized to the DiCo-specific IgG (Figure 5A) as well as to allele-specific IgG (Figure 5B). The results clearly show that for both, DiCo-specific IgG, as well as strain-specific IgG, there is no significant difference between the different groups regarding the IC_50_ values observed for each of the three different strains. It is also obvious that the mean IC_50_ values (between 120 and 180 µg/ml) observed for the DiCo-specific IgG are in the expected range and do not differ significantly for the three strains, which proves the induction of a cross-strain-specific immune response. The differences between DiCo and single allele-specific IC_50_ values are proportional to the fraction of allele-specific IgG within the DiCo-specific antibody response (Figure 4B).

## Discussion

A preparation consisting of recombinant *Pf*AMA1 and its peptide ligand *Pf*Ron2L was recently shown to achieve greater efficacy than *Pf*AMA1 alone in an *in vitro* parasite GIA using *P. falciparum* and to protect mice from a lethal challenge with *P. yoelii* (35). We, therefore, tested the same strategy to determine whether it could enhance the potency of antibody responses when combining recombinant DiCo variants of *Pf*AMA1 with *Pf*Ron2L. The available epidemiological (46), preclinical (14), and clinical data (47, 48) clearly show that both greater potency (by increasing specificity or immunogenicity) and improved cross-strain coverage are required to develop an efficient *Pf*AMA1-based blood-stage vaccine (19, 49). We immunized three groups of four rabbits with a balanced mixture of the three DiCo variants (D), or DiCo-Mix and *Pf*Ron2L at different injection sites (D + R), or the DiCo-Mix in complex with *Pf*Ron2L (C), and conducted a detailed quantitative and qualitative analysis of the resulting immune IgG, using different ELISA formats, SPR-based binding assays and parasite GIAs.

In agreement with our earlier DiCo-based studies (37, 44) and those reported by other groups (19, 45), we observed balanced IC_50_ values (~150 μg/ml) against different strains. This is in contrast to using single alleles for immunization, where the IC_50_ values in GIA are significantly higher (greater than twofold) for heterologous compared to homologous (vaccine-like) strains (18).

On one hand, comparison among the three immunization groups (D, D + R, and C) showed that co-immunization with *Pf*Ron2L [either at a different injection site (D + R) or in a complex (C)] does not reduce the ability of the DiCo-Mix to induce cross-strain parasite inhibitory responses. On the other hand, there was no significant difference in IC_50_ values between the DiCo–*Pf*Ron2L complex group and the other two groups, as reported for homologous GIAs using *P. falciparum* and a lethal challenge using *P. yoelii* following the immunization of mice with single-allele AMA1–Ron2L complexes (35).

Even though these results appear contradictory, there may be a common explanation. The authors of the abovementioned study provided evidence that the improved efficacy of the AMA1–Ron2L complex relies on antibody responses, and that antibodies against certain variable loops surrounding the Ron2-binding pocket play an important role in this scenario (35). Parasite growth-inhibitory antibodies interfering with the AMA1–Ron2L interaction by recognizing these variable (or even hypervariable) loops are most probably strain specific and, therefore, less favorable when aiming for cross-strain efficacy. This is further illustrated by the monoclonal antibody 1F9 (50), a murine antibody raised by immunization with *Pf*AMA1-3D7, which interferes with the *Pf*AMA1–*Pf*Ron2 interaction by binding to a reduction-sensitive epitope including the most polymorphic residue of the antigen. Point mutations at this residue, such as those found in alleles *Pf*AMA1-HB3 and *Pf*AMA1-W2_mef_, prevent 1F9 binding to the corresponding alleles and eliminate growth inhibitory activity against these strains (28). Immunization with multiple *Pf*AMA1 alleles improved cross-strain efficacy by increasing the proportion of conserved face-specific antibodies that recognize epitopes shared by the majority or even all known *P. falciparum* strains (14, 15, 17, 18). Taken together, these experiments suggest that at least four (18) or five (17) different alleles must be combined to induce cross-strain-specific responses covering diverse naturally occurring strains.

Each additional allele provided as a recombinant protein in the context of a vaccine formulation adds to the costs and complexity of process development, manufacturing, and regulatory approval, so, three DiCo variants have been designed to cover the allelic diversity of *Pf*AMA1 comprehensively using the smallest number of recombinant proteins (19). As discussed above for the conventional multi-allele approach (14, 15, 17, 18), the DiCo approach successfully increases the induction of conserved region-specific antibodies by dilution of the strain-specific variable epitopes (51). Alternative approaches that drive the immune response toward conserved *Pf*AMA1 epitopes include the immunodampening of the hypervariable loop Id (52) as well as glycan masking of the variable regions (Boes et al., in preparation). If the improved potency of the AMA1–Ron2L complex results from the induction of antibodies targeting variable loops near the Ron2-binding pocket that undergo conformational changes when Ron2 binds (35), then a strategy favoring cross-strain specific epitopes by overrepresentation of the conserved regions may reduce the induction of such antibodies below effective concentrations, which is probably why the IC_50_-values we observed could not be improved by the combination of DiCo-Mix with *Pf*Ron2L. Alternatively, the artificial mixed allele design approach of the *Pf*AMA1 DiCo variants could affect the conformational changes normally induced by *Pf*Ron2-binding in the variable loop region of natural *Pf*AMA1 alleles and thus fail to induce efficacious antibodies. Although these explanations are speculative and require conformational studies, they highlights the complexity associated with *Pf*AMA1 as a vaccine target.

In our setting, co-formulation with the *Pf*Ron2L peptide did not improve the *in vitro* efficacy of the DiCo-Mix, a vaccine that aims for cross-strain coverage by eliciting higher levels of constant region-specific antibodies. However, our results provide additional insight into DiCo-specific antibody responses. The competition experiment revealed that single *Pf*AMA1 alleles neutralize between 65% (*Pf*AMA1-3D7) and 75% (*Pf*AMA1-FCR3) of DiCo-specific immune IgG. Whereas conserved region-specific immune IgG will be neutralized by all alleles, antibodies against polymorphic, strain-specific regions will only be neutralized by alleles that present the corresponding epitopes. The observed trend toward different degrees of competition of the three alleles (*Pf*3D7, *Pf*FCR3, and *Pf*HB3), although not statistically significant, most probably reflects the ability of the DiCo-Mix to induce corresponding strain-specific IgG in addition to commonly neutralizing cross-strain-specific antibodies, given that constant region-specific antibodies will be neutralized equally by all *Pf*AMA1 alleles and DiCo variants. The trend indicates that DiCo-specific immune IgG may contain a lower proportion of strain-specific IgG directed toward *Pf*AMA1*-*3D7 than *Pf*AMA1*-*FCR3 or *Pf*AMA1*-*HB3. The comparison of GIA IC_50_ values (~125 μg/ml) for *Pf*AMA1-3D7-specific antibodies in immune IgG preparations derived from the three immunization groups (D, D + R, and C) and the corresponding IC_50_ values for *Pf*AMA1-3D7-specific antibodies (~40 μg/ml) generated by single-allele immunization with *Pf*AMA1-3D7 (38) suggests that DiCo-derived *Pf*AMA1-3D7-specific antibodies have a lower *in vitro* efficacy. This contrasts with the results of a *Pf*AMA1 multi-allele study in which identical IC_50_ values were observed for affinity-purified *Pf*AMA1*-*3D7-specific antibodies derived from rabbits immunized with either single-allele *Pf*AMA1*-*3D7 or with a mixture (Quadvax) of the four different *Pf*AMA1 alleles 3D7, FVO, HB3, and W2_mef_ (18). Even though Quadvax seems to induce predominantly conserved region-specific antibodies, it is possible that the presence of *Pf*AMA1-3D7 within the vaccine formulation leads to the induction of potent, allele-specific growth inhibitory antibodies, which account for this difference. Looking at an alignment of sequences featuring the three DiCo variants, as well as the three alleles *Pf*AMA1*-*3D7, *Pf*AMA1*-*FCR3, and *Pf*AMA1*-*HB3 (Figure S1 in Supplementary Material), we find a glutamic acid residue (E197) in the hypervariable loop Id (33), which is not present in any of the three DiCo variants or strains *Pf*AMA1*-*3D7, *Pf*AMA1*-*FCR3, and *Pf*AMA1*-*HB3. E197, the most polymorphic residue in *Pf*AMA1, is a key residue within an epitope targeted by the *Pf*3D7 growth-inhibitory monoclonal antibody 1F9 (28). Replacing this residue with random amino acids or those found in other allelic variants of *Pf*AMA1 abolishes 1F9 binding and consequently growth-inhibitory activity. Another hint that antibodies directed to the Id loop may play an essential role was shown by replacing all polymorphic residues within the Id loop with alanine, aiming to reduce Id-specific reactivity and generate improved cross-strain-specific responses targeting the conserved region (52). The improved cross-strain efficacy was achieved at the cost of reduced overall growth-inhibitory activity for the homologous strain, which is analogous to our observations. All these results and conclusions together suggest that it would be more promising to work on the induction of highly potent or maximally cross-strain-specific antibodies by investing resources into concepts that target these goals separately, and, if reasonable, combine them once both approaches show promising improvements beyond the current state of the art.

## Ethics Statement

Rabbits were housed, immunized, and sampled by Biogenes GmbH (Berlin, Germany) according to national animal welfare regulations. The animal facilities and protocols were reviewed and approved by: Landesamt für Landwirtschaft, Lebensmittelsicherheit, und Fischerei MecklenburgVorpommern (LALLF M-V) (Approval No: 7221.3-2-030-13).

## Author Contributions

AB and HS conceived the study, performed the experiments, analyzed the data, and wrote the manuscript. RF performed and analyzed the GIA experiments and contributed to data analysis and writing the manuscript. AR, SS, and RFi conceived the overall study design and contributed to writing the manuscript. All authors read and approved the final manuscript.

## Conflict of Interest Statement

The authors declare that the research was conducted in the absence of any commercial or financial relationships that could be construed as a potential conflict of interest.
