# Supplementary material for: Immunization with the Malaria Diversity-Covering Blood-Stage Vaccine Candidate Plasmodium falciparum Apical Membrane Antigen 1 DiCo in Complex with Its Natural Ligand PfRon2 Does Not Improve the In Vitro Efficacy
Source: Front Immunol. 2017 Jun 27;8:743. doi: 10.3389/fimmu.2017.00743 (PMC5484772; doi:10.3389/fimmu.2017.00743)
Supplement: Supplementary file 1 [file data_sheet_1.docx]

DiCo1 QNYWEHPYQKSDVYHPINEHREHPKEYEYPLHQEHTYQQEDSGEDENTLQHAYPIDHEGA

DiCo2 QNYWEHPYQKSDVYHPINEHREHPKEYEYPLHQEHTYQQEDSGEDENTLQHAYPIDHEGA

DiCo3 QNYWEHPYQKSDVYHPINEHREHPKEYEYPLHQEHTYQQEDSGEDENTLQHAYPIDHEGA

3D7 QNYWEHPYQNSDVYRPINEHREHPKEYEYPLHQEHTYQQEDSGEDENTLQHAYPIDHEGA

7G8 QNYWEHPYQKSDVYHPINEHREHSKEYEYPLHQEHTYQQEDSGEDENTLQHAYPIDHEGA

Dd2 QNYWEHPYQKSDVYHPINEHREHPKEYQYPLHQEHTYQQEDSGEDENTLQHAYPIDHEGA

FCR3 QNYWEHPYQKSDVYHPINEHREHPKEYEYPLHQEHTYQQEDSGEDENTLQHAYPIDHEGA

HB3 QNYWEHPYQNSDVYRPINEHREHPKEYEYPLHQEHTYQQEDSGEDENTLQHAYPIDHEGA

RO33 QNYWEHPYQKSDVYHPINEHREHPKEYEYPLHQEHTYQQEDSGEDENTLQHAYPIDHEGA

*********:****:******** ***:********************************

DiCo1 EPAPQEQNLFSSIEIVERSNYMGNPWTEYMAKYDIEEVHGSGIRVDLGEDAEVAGTQYRL

DiCo2 EPAPQEQNLFSSIEIVERSNYMGNPWTEYMAKYDIEEVHGSGIRVDLGEDAEVAGTQYRL

DiCo3 EPAPQEQNLFSSIEIVERSNYMGNPWTEYMAKYDIEEVHGSGIRVDLGEDAEVAGTQYRL

3D7 EPAPQEQNLFSSIEIVERSNYMGNPWTEYMAKYDIEEVHGSGIRVDLGEDAEVAGTQYRL

7G8 EPAPQEQNLFSSIEIVERSNYMGNPWTEYMAKYDIEEVHGSGIRVDLGEDAEVAGTQYRL

Dd2 EPAPQEQNLFSSIEIVERSNYMGNPWTEYMAKYDIEEVHGSGIRVDLGEDAEVAGTQYRL

FCR3 EPAPQEQNLFSSIEIVERSNYMGNPWTEYMAKYDIEEVHGSGIRVDLGEDAEVAGTQYRL

HB3 EPAPQEQNLFSSIEIVERSNYMGNPWTEYMAKYDIEKVHGSGIRVDLGEDAEVAGTQYRL

RO33 EPAPQEQNLFSSIEIVERSNYMGNPWTEYMAKYDIEEVHGSGIRVDLGEDAEVAGTQYRL

************************************:***********************

DiCo1 PSGKCPVFGKGIIIENSQTTFLTPVATENQDLKDGGFAFPPTKPLMSPMTLDQMRHFYKD

DiCo2 PSGKCPVFGKGIIIENSQTTFLKPVATGNQDLKDGGFAFPPTNPLISPMTLNGMRDFYKN

DiCo3 PSGKCPVFGKGIIIENSKTTFLTPVATENQDLKDGGFAFPPTEPLMSPMTLDDMRDLYKD

3D7 PSGKCPVFGKGIIIENSNTTFLTPVATGNQYLKDGGFAFPPTEPLMSPMTLD**E**MRHFYKD

7G8 PSGKCPVFGKGIIIENSNTTFLKPVATGNQDLKDGGFAFPPTNPLISPMTLDHMRDFYKN

Dd2 PSGKCPVFGKGIIIENSNTTFLTPVATGNQYLKDGGFAFPPTKPLMSPMTLDDMRLLYKD

FCR3 PSGKCPVFGKGIIIENSNTTFLKPVATGNQDLKDGGFAFPPTNPLISPMTLNGMRDFYKN

HB3 PSGKCPVFGKGIIIENSKTTFLTPVATENQDLKDGGFAFPPTEPLISPMTLDQMRHLYKD

RO33 PSGKCPVFGKGIIIENSNTTFLKPVATGNQDLKDGGFAFPPTEPLISPMTLNGMRDFYKN

*****************:****.**** ** ***********:**:*****: ** :**:

DiCo1 NEYVKNLDELTLCSRHAGNMNPDNDKNSNYKYPAVYDDKDKKCHILYIAAQENNGPRYCN

DiCo2 NEYVKNLDELTLCSRHAGNMNPDNDENSNYKYPAVYDYNDKKCHILYIAAQENNGPRYCN

DiCo3 NKYVKNLDELTLCSRHAGNMIPDNDKNSNYKYPAVYDYEDKKCHILYIAAQENNGPRYCN

3D7 NKYVKNLDELTLCSRHAGNMIPDNDKNSNYKYPAVYDDKDKKCHILYIAAQENNGPRYCN

7G8 NEYVKNLDELTLCSRHAGNMNPDNDKNSNYKYPAVYDYNDKKCHILYIAAQENNGPRYCN

Dd2 NEDVKNLDELTLCSRHAGNMNPDNDKNSNYKYPAVYDYNDKKCHILYIAAQENNGPRYCN

FCR3 NEYVKNLDELTLCSRHAGNMNPDNDKNSNYKYPAVYDYNDKKCHILYIAAQENNGPRYCN

HB3 NEYVKNLDELTLCSRHAGNMNPDNDKNSNYKYPAVYDYEDKKCHILYIAAQENNGPRYCN

RO33 NEYVKNLDELTLCSRHAGNMNPDKDENSNYKYPAVYDDKDKKCHILYIAAQENNGPRYCN

*: ***************** **:*:*********** :*********************

DiCo1 KDESKRNSMFCFRPAKDKSFQNYVYLSKNVVDNWEKVCPRKNLENAKFGLWVDGNCEDIP

DiCo2 KDESKRNSMFCFRPAKDKLFENYVYLSKNVVHNWEEVCPRKNLENAKFGLWVDGNCEDIP

DiCo3 KDQSKRNSMFCFRPAKDISFQNYVYLSKNVVDNWEKVCPRKNLQNAKFGLWVDGNCEDIP

3D7 KDESKRNSMFCFRPAKDISFQNYTYLSKNVVDNWEKVCPRKNLQNAKFGLWVDGNCEDIP

7G8 KDESKRNSMFCFRPAKDKSFQNYTYLSKNVVDNWEKVCPRKNLENAKFGLWVDGNCEDIP

Dd2 KDESKRNSMFCFRPAKDKSFQNYTYLSKNVVDNWEEVCPRKNLENAKFGLWVDGNCEDIP

FCR3 KDQSKRNSMFCFRPAKDKLFENYTYLSKNVVDNWEEVCPRKNLENAKFGLWVDGNCEDIP

HB3 KDESKRNSMFCFRPAKDKLFENYTYLSKNVVDNWEEVCPRKNLENAKFGLWVDGNCEDIP

RO33 KDESKRNSMFCFRPAKDKSFQNYTYLSKNVVDNWEKVCPRKNLENAKFGLWVDGNCEDIP

**:************** *:**.*******.***:*******:****************

DiCo1 HVNEFSANDLFECNKLVFELSASDQPKQYEQHLTDYEKIKEGFKNKNADMIRSAFLPTGA

DiCo2 HVNEFSANDLFECNKLVFELSASDQPKQYEQHLTDYEKIKEGFKNKNADMIRSAFLPTGA

DiCo3 HVNEFSAIDLFECNKLVFELSASDQPKQYEQHLTDYEKIKEGFKNKNADMIRSAFLPTGA

3D7 HVNEFPAIDLFECNKLVFELSASDQPKQYEQHLTDYEKIKEGFKNKNASMIKSAFLPTGA

7G8 HVNEFSANDLFECNKLVFELSASDQPKQYEQHLTDYEKIKEGFKNKNASMIKSAFLPTGA

Dd2 HVNEFSANDLFECNKLVFELSASDQPKQYEQHLTDYEKIKEGFKNKNASMIKSAFLPTGA

FCR3 HVNEFSANDLFECNKLVFELSASDQPKQYEQHLTDYEKIKEGFKNKNASMIKSAFLPTGA

HB3 HVNEFSANDLFECNKLVFELSASDQPKQYEQHLTDYEKIKEGFKNKNASMIKSAFLPTGA

RO33 HVNEFSANDLFECNKLVFELSASDQPKQYEQHLTDYEKIKEGFKNKNASMIKSAFLPTGA

***** * ****************************************.**:********

DiCo1 FKADRYKSHGKGYNWGNYNRKTQKCEIFNVKPTCLINDKSYIATTALSHPIEVEHNFPCS

DiCo2 FKADRYKSRGKGYNWGNYNRKTQKCEIFNVKPTCLINDKSYIATTALSHPIEVENNFPCS

DiCo3 FKADRYKSHGKGYNWGNYNTETQKCEIFNVKPTCLINDKSYIATTALSHPNEVEHNFPCS

3D7 FKADRYKSHGKGYNWGNYNTETQKCEIFNVKPTCLINNSSYIATTALSHPIEVENNFPCS

7G8 FKADRYKSRGKGYNWGNYNRKTQKCEIFNVKPTCLINNSSYIATTALSHPNEVEHNFPCS

Dd2 FKADRYKSHGKGYNWGNYNRKTQKCEIFNVKPTCLINNSSYIATTALSHPIEVEHNFPCS

FCR3 FKADRYKSHGKGYNWGNYNRETQKCEIFNVKPTCLINNSSYIATTALSHPIEVEHNFPCS

HB3 FKADRYKSRGKGYNWGNYNTETQKCEIFNVKPTCLINNSSYIATTALSHPNEVENNFPCS

RO33 FKADRYKSHGRGYNWGNYNRKTQKCEIFNVKPTCLINNSSYIATTALSHPIEVENNFPCS

********:*:******** :****************:.*********** ***:*****

DiCo1 LYKDEIKKEIERESKRIKLNDNDDEGNKKIIAPRIFISDDKDSLKCPCDPEIVSQSTCNF

DiCo2 LYKNEIMKEIERESKRIKLNDNDDEGNKKIIAPRIFISDDKDSLKCPCDPEMVSQSTCRF

DiCo3 LYKDEIKKEIERESKRIKLNDNDDEGNKKIIAPRIFISDDIDSLKCPCAPEIVSQSTCNF

3D7 LYKDEIMKEIERESKRIKLNDNDDEGNKKIIAPRIFISDDKDSLKCPCDPEMVSNSTCRF

7G8 LYKDEIKKEIERESKRIKLNDNDDEGNKKIIAPRIFISDDIDSLKCPCDPEIVSNSTCNF

Dd2 LYKDEIKKEIERESKRIKLNDNDDEGNKKIIAPRIFISDDIDSLKCPCDPEIVSNSTCNF

FCR3 LYKDEIKKEIERESKRIKLNDNDDEGNKKIIAPRIFISDDKDSLKCPCDPEMVSNSTCRF

HB3 LYKDEIKKEIERESKRIKLNDNDDEGNKKIIAPRIFISDDKDSLKCPCDPEIVSNSTCNF

RO33 LYKNEIMKEIERESKRIKLNDNDDEGNKKIIAPRIFISDDKDSLKCPCDPEIVSNSTCNF

***:** ********************************* ******* **:**:***.*

DiCo1 FVCKCVEKRAEVTSNNEVVVKEEYKDEYADIPEHKPTYDKM

DiCo2 FVCKCVERRAEVTSNNEVVVKEEYKDEYADIPEHKPTYDNM

DiCo3 FVCKCVEKRAEVTSNNEVVVKEEYKDEYADIPEHKPTYDKM

3D7 FVCKCVERRAEVTSNNEVVVKEEYKDEYADIPEHKPTYDKM

7G8 FVCKCVEKRAEVTSNNEVVVKEEYKDEYADIPEHKPTYDKM

Dd2 FVCKCVEKRAEVTSNNEVVVKEEYKDEYADIPEHKPTYDKM

FCR3 FVCKCVERRAEVTSNNEVVVKEEYKDEYADIPEHKPTYDNM

HB3 FVCKCVEKRAEVTSNNEVVVKEEYKDEYADIPEHKPTYDNM

RO33 FVCKCVEKRAEVTSNNEVVVKEEYKDEYADIPEHKPTYDKM

*******:*******************************:*

|  | DiCo1 | DiCo2 | DiCo3 | 3D7 | 7G8 | Dd2 | FCR3 | HB3 | RO33 |
| --- | --- | --- | --- | --- | --- | --- | --- | --- | --- |
| DiCo1 | - | 95 | 96 | 95 | 96 | 96 | 95 | 95 | 95 |
| DiCo2 | 95 | - | 93 | 93 | 95 | 94 | 97 | 94 | 95 |
| DiCo3 | 96 | 93 | - | 95 | 95 | 95 | 93 | 95 | 93 |
| 3D7 | 95 | 93 | 95 | - | 94 | 95 | 95 | 95 | 95 |
| 7G8 | 96 | 95 | 95 | 94 | - | 97 | 97 | 95 | 97 |
| Dd2 | 96 | 94 | 95 | 95 | 97 | - | 96 | 95 | 95 |
| FCR3 | 95 | 97 | 93 | 95 | 97 | 96 | - | 95 | 96 |
| HB3 | 95 | 94 | 95 | 95 | 95 | 95 | 95 | - | 95 |
| RO33 | 95 | 95 | 93 | 95 | 97 | 95 | 96 | 95 | - |

**Supplementary material S1:** Sequence alignment of the three DiCo variants [19] and six *Pf*AMA1 alleles (3D7 Genbank: XM_001347979, 7G8 Genbank: EU586371, Dd2 Genbank: EU586381, FCR3 Genbank: EU586390, HB3 Genbank: EU586393 and RO33 Genbank: AB715734). The annotation of the Id loop (underlined) was according to Bai *et al.* [33]. Glutamin (E197) in the amino acid sequence of *Pf*AMA1-3D7 is indicated in pink and bold. The similarity table shows pairwise similarity scores of *Pf*AMA1 alleles and the three DiCo variants.
